# SARS-CoV-2 antibodies seroprevalence in dogs from France using ELISA and an automated western blotting assay

**DOI:** 10.1016/j.onehlt.2021.100293

**Published:** 2021-07-18

**Authors:** Younes Laidoudi, Youssouf Sereme, Hacène Medkour, Stéphanie Watier-Grillot, Pierre Scandola, Jacques Ginesta, Virginie Andréo, Claire Labarde, Loïc Comtet, Philippe Pourquier, Didier Raoult, Jean-Lou Marié, Bernard Davoust

**Affiliations:** a Aix Marseille Univ, IRD, AP-HM, MEPHI, Marseille, France; b IHU Méditerranée Infection, Marseille, France; c French Military Health Service, Animal Epidemiology Expert Group, Tours, France; d French Army Center for Epidemiology and Public Health, Marseille, France; e 24^th^ Veterinary Group, Suippes, France; f 26^th^ Veterinary Group, Gramat, France; g 1^th^ Veterinary Group, Toulon, France; h Innovative Diagnostics, Grabels, France

**Keywords:** COVID-19, SARS-CoV-2, Serology, Dog, Epidemiosurveillance, One health

## Abstract

Dogs are occasionally susceptible to SARS-CoV-2, developing few or no clinical signs.

Epidemiological surveillance of SARS-CoV-2 in dogs requires testing to distinguish it from other canine coronaviruses. In the last year, significant advances have been made in the diagnosis of SARS-CoV-2, allowing its surveillance in both human and animal populations. Here, using ELISA and automated western blotting (AWB) assays, we performed a longitudinal study on 809 apparently healthy dogs from different regions of France to investigate anti-SARS-CoV-2 antibodies. There were three main groups: (i) 356 dogs sampled once before the pandemic, (ii) 235 dogs sampled once during the pandemic, and (iii) 218 dogs, including 82 dogs sampled twice (before and during the pandemic), 125 dogs sampled twice during the pandemic and 11 dogs sampled three times (once before and twice during the pandemic). Using ELISA, seroprevalence was significantly higher during the pandemic [5.5% (25/453)] than during the pre-pandemic period [1.1% (5/449)]. Among the 218 dogs sampled twice, at least 8 ELISA-seroconversions were observed. ELISA positive pre-pandemic sera were not confirmed in serial tests by AWB, indicating possible ELISA cross-reactivity, probably with other canine coronaviruses. A significant difference was observed between these two serological tests (Q = 88, *p* = 0.008). A clear correlation was observed between SARS-CoV-2 seroprevalence in dogs and the incidence of SARS-CoV-2 infection in human population from the same area. AWB could be used as a second line assay to confirm the doubtful and discrepant ELISA results in dogs. Our results confirm the previous experimental models regarding the susceptibility of dogs to SARS-CoV-2, suggesting that viral transmission from and between dogs is weak or absent. However, the new variants with multiple mutations could adapt to dogs; this hypothesis cannot be ruled out in the absence of genomic data on SARS-CoV-2 from dogs.

## 1 Introduction

Severe Acute Respiratory Syndrome, caused by SARS-CoV-2 coronavirus, a novel emergent variant involved in epidemic disease, was first identified in November 2019 in Wuhan city (Hubei province), China [1,2]. A few months later, the World Health Organization declared a global pandemic. By early July 2021, more than 183 million cases and 3.96 million deaths had been recorded worldwide [3]. In France, the first human cases were diagnosed in late January 2020. At the beginning of July 2021, the cumulative incidence for France reached almost 5.84 million, including 111,297 deaths [3,4].

Phylogenetically, SARS-CoV-2 is closely related to SARS CoV (or SARS-CoV-1), which was already involved in the 2003 epidemic, and to BatCoV RaTG13, a Betacoronavirus naturally occurring in bats [5,6]. The scientific community assumes that SARS-CoV-2 has a zoonotic origin from bats, while the intermediate host between bats and humans is not yet known [2,5,6]. Due to the presence of specific receptors for SARS-CoV-2 virus in the respiratory tract of mustelids (i.e. ferrets and minks), they are the most susceptible species under both experimental and natural conditions [7]. Globally, coronaviruses are widely distributed in animal fauna (i.e. birds, pigs, ruminants, dogs, cats, etc.) [2,[5], [6], [7], [8], [9], [10], [11]]. Since the 1970s, Alpha and Betacoronavirus have been in the forefront as the causative agents of canine enteritic coronavirus (CECoV) and respiratory coronavirus (CRCoV), respectively [12,13]. However, dogs are occasionally susceptible to SARS-CoV-2 with about one hundred cases diagnosed worldwide by specific analysis (RT-qPCR) in Hong Kong, USA, Japan, Argentina and Italy by the end of 2020 [9,14,15]. Dogs infected with SARS-CoV-2 have few or no clinical signs [14]. Epidemiological surveillance of SARS-CoV-2 in dogs requires reliable serological methods to distinguish SARS-CoV-2 from other canine coronaviruses. In the last year, advances have been made in the diagnosis of SARS-CoV-2, and monitoring the circulation of the virus in human and animal populations. Here, we performed a longitudinal study of the seroprevalence of SARS-CoV-2 in apparently healthy dogs from different regions of France to highlight the epidemiological role of these animals in the current SARS-CoV-2 pandemic.

## 2 Materials and methods

### 2.1 Dogs

A total of 809 dogs from France were included in this study (i.e. Bouches-du-Rhône, Marne, Lot, Var, Vaucluse, Corsica and French Guiana), of which 449 serum samples were collected before the SARS-CoV-2 pandemic (from 2006 to January 2020) and 453 during the pandemic (from February 2020 to February 2021). Of these, 559 (69%) were military working dogs (MWD), mainly male Belgian shepherds and German shepherds, aged one to ten years, and 250 (31%) were companion dogs (adult dogs of both sexes, mostly living in shelters). The dogs were allocated into three groups: (i) 356 dogs were sampled once before the pandemic, (ii) 235 dogs were sampled once during the pandemic and (iii) 218 dogs, including 82 dogs sampled twice (before and during the pandemic), 125 dogs sampled twice during the pandemic and 11 dogs sampled three times (once before and twice during the pandemic). A total of 1038 blood samples were collected using a 3.5 mL vacuum tube with serum separation gel. Canine sera were harvested and stored at −20 °C or + 4 °C until analysis.

Serum samples were taken in veterinary clinics for screening by veterinary doctors and with the agreement of the owners of the dogs.

### 2.2 ELISA assay

All sera were subjected to screening for antibodies to SARS-CoV-2 using ID Screen® SARS-CoV-2 Double Antigen Multi-species (Innovative Diagnostics, Grabels, France) according to the manufacturer's instructions. The test consists of an ELISA (enzyme-linked immunosorbent assay), that detects antibodies to the major nucleocapsid protein of SARS-CoV-2 on multispecies (i.e. minks, ferrets, cats, dogs, cattle, sheep, goats, horses and all other receptive species) with a specificity range of 97.8% to 100% as reported by the manufacturer (Supplementary data 1). Plates were sensitized with a purified recombinant N antigen. Optical density (OD) was measured at 450 nm using Multiskan GO software (Thermo Scientific, Waltham, MA, USA). The assay was validated when the optical density of positive control (OD_PC_) was ≥0.35 and a mean of positive control (OD_PC_) to negative control (OD_NC_) control ratio was greater than three. The optical density of each sample (OD_N_) was used to calculate the S/P ratio value (expressed as %) where S/*P* = 100 * (OD_N_ - OD_NC_)/ (OD_PC_ - OD_NC_). Samples tested by ELISA were considered positive if the S/P ratio was greater than 60% and doubtful when the P/S ratio ranged between 50 and 60%, while samples displaying an S/P score lower than 50% in ELISA were considered negative.

### 2.3 SARS-CoV-2 antigen preparation and automated western blotting (AWB) assay

The SARS-CoV-2 IHUMI2 strain (lineage 20a) was used to produce of SARS-CoV-2 specific antigens as previously described [16]. Briefly, virions were purified and harvested from in vitro infected cells and then fractionated with TS buffer (7 M Urea, 2 M Thiourea, 4% Chaps) to release SARS-CoV-2 antigens. The released antigens were concentrated using the Amicon 3 kDa filter (Merck KGaA, Darmstadt, Germany) before being used in the Automated Western Blotting (AWB) assay [16,17].

The Jess™ Simple Western automated nano-immunoassay system (ProteinSimple, San Jose, CA, USA, a Bio-Techne Brand), a capillary-based size separation of proteins [16] was used with an internal system control to evaluate the absolute serological response to viral antigens from all ELISA-positive samples. Canine sera were processed according to the manufacturer's standard method for the 12–230-kDa Jessseparation module (SM-W004). The Edouard's protocol [16] was adapted for the detection of canine antibodies to SARS-CoV-2. Briefly, a mixture of SARS-CoV-2 antigens, fluorescent molecular weight markers and 400 mM dithiothreitol (Protein Simple) was prepared at final concentration of 0.25 μg/μl, and then denatured at 95 °C for 5 min. Migration of viral proteins through the separation matrix was performed at 375 V for both SARS-CoV-2 antigens and Ladder (12–230-kDa PS-ST01EZ). The separated proteins were immobilized using the photoactivated capture chemistry within the ProteinSimple proprietary [16]. Subsequently, 1:2 diluted dog sera were incubated for 60 min followed by a wash step and a 30 min incubation within a multi-species HRP-conjugated anti-Fc fragment of IgG/IgM/IgA antibodies (Innovative Diagnostics, Grabels, France). Peroxide/luminol-S (ProteinSimple) was used for chemiluminescent revelation. The Compass Simple Western software (version 5.0.1, ProteinSimple) was used for the automatic calculation of the heights (chemiluminescence intensity), area and signal/noise ratio as well as to capture the digital image of the capillary chemiluminescence.

### 2.4 Statistical analysis

Only one serum sample from each dog at each time-point (during and pre-pandemic) was considered in the calculation of infection rate. Comparison between dog's populations was performed using Fisher's exact and Chi-squared tests. Mc Nemar's test was used to compare between ELISA and AWB assays. The exact *p*-value was computed, and the significant difference was considered at a p-value ≤0.05. All statistical analysis were performed using Addinsoft software (XLSTAT 2018: Data Analysis and Statistical Solution for Microsoft Excel, Paris, France).

## 3 Results

### 3.1 ELISA antibody detection

Of the 449 sera sampled before the pandemic, 4 (0.9%) were ELISA positive and 1 (0.2%) was inconclusive. While of the 453 sera collected during the pandemic, 22 (4.8%) were positive and 3 (0.6%) were classified as doubtful. The infection rate was significantly higher during the pandemic than in the pre-pandemic period (Table 1). In addition, at least 8 ELISA-seroconversions were observed among the 218 dogs during the pandemic (Table 2). During the pandemic, a total of 17 (4.3%) out of 397 MWD and 7 (12.5%) out of 56 companion dogs reacted in the ELISA test, corresponding to a significant difference (Khi2 = 6.61 - *p* ≤ 0.02) between these two populations. Fourteen (11.1%) of 126 dogs sampled in February 2021 from the South-East area scored positive. A lower prevalence of 3.1% (3/95) was found in the South-West than in the South-East (Khi2 = 4.7 - *p* ≤ 0.05) (Table 3).

**Table 1:** Seroprevalence of SARS-CoV-2 antibodies, detected with a double antigen ELISA test, in dogs from France before and during the COVID-19 pandemic.

	Sample description	No. of sera	No. of positive (%)	No. of doubtful (%)	No. of sera reacted with ELISA (%)
Total sera*	Before the pandemic	449	4 (0.9%)	1 (0.2%)	5 (1.1%)
	During the pandemic	453	22 (4.8%)	3 (0.6%)	25 (5.5%)
	Statistics		Khi2 = 13.56; *p* < 0.001		
Sera sampled one time	Before the pandemic	356	3 (0.8%)	0 (0%)	3 (0.8%)
	During the pandemic	235	14 (5.9%)	0 (0%)	14 (5.9%)
	Statistics		Khi2 = 13.26; p < 0.001		
Activity of the investigated dogs during the pandemic	MWD	397	15 (3.7%)	2 (0.5%)	17 (4.3%)
	SD and PD	56	7 (12.5%)	0 (0%)	7 (12.5%)
	Statistics		Khi2 = 6.61; *p* < 0.02		

SD: shelter dogs, MWD: military working dogs, PD: pet dogs, *: only one serum sample from each dog at each time-period.

**Table 2:** Individual positive results of serological detection of SARS-CoV-2 infection by the double antigen ELISA test (*N* = 28 dogs).

	Serum id.	Dog id.	Dog category	Location department	Date of sample	ELISA OD	(S/P) %	ELISA result	AWB result
Dogs collected once before the pandemic (N = 356)	D699	D1	SD	French Guiana	01.2016	0.558	231.1	Positive	Negative
	D681	D2	SD	Corsica	03.2018	1.012	424.3	Positive	Negative
	D662	D3	MWD	Bouches-du-Rhône	10.2018	0.214	84.7	Positive	Not tested
Dogs collected once during the pandemic (N = 235)	D95	D4	MWD	Marne	06.2020	3.89	278.1	Positive	**Positive**
	D132	D5	MWD		09.2020	0.901	61.2	Positive	Negative
	D306	D6	SD	Bouches-du-Rhône	02.2021	0.78	166.8	Positive	Negative
	D307	D7	SD		02.2021	0.764	162.9	Positive	Negative
	D312	D8	SD		02.2021	0.62	127.8	Positive	**Positive**
	D697	D9	SD		02.2021	0.197	77.4	Positive	**Positive**
	D357	D10	SD		02.2021	0.739	156.8	Positive	**Positive**
	D358	D11	SD		02.2021	0.52	103.4	Positive	**Positive**
	D400	D12	MWD		02.2021	1.648	435.2	Positive	**Positive**
	D441	D13	MWD		02.2021	0.82	207.7	Positive	**Positive**
	D568	D14	MWD	Lot	02.2021	0.332	49.0	Positive	**Positive**
	D627	D15	MWD		02.2021	0.41	61.2	Positive	Negative
	D646	D16	MWD		02.2021	0.421	62.9	Positive	**Positive**
	D438	D17	MWD	Bouches-du-Rhône	02.2021	0.29	62.1	Positive	Negative
Dogs collected twice, once before and once during the pandemic (82)	D560	D18	MWD	Bouches-du-Rhône	06.2018	0.043	0.7	Negative	Not tested
	CR10		MWD		10.2020	0.344	55.3	Doubtful	Not tested
	D211	D19	MWD	Marne	11.2019	0.065	1.4	Negative	Not tested
	D210		MWD		05.2020	1.166	96.0	Positive	Negative
	D23	D20	MWD		01.2020	1.31	83.0	Positive	**Positive**
	D24		MWD		06.2020	1.373	87.2	Positive	**Positive**
Dogs collected twice during the pandemic (*N* = 125)	D229	D21	MWD	Marne	03.2020	0.21	13.8	Negative	Negative
	D228		MWD		07.2020	0.821	66.4	Positive	**Positive**
	D57	D22	MWD		04.2020	0.435	25.0	Negative	Not tested
	D58		MWD		09.2020	0.89	55.2	Doubtful	**Positive**
	D96	D23	MWD		06.2020	0.292	17.0	Negative	Not tested
	D97		MWD		10.2020	0.704	46.9	Negative	Not tested
	D175	D24	MWD		07.2020	0.069	0.8	Negative	Negative
	D696		MWD		01.2021	0.106	38.7	Negative	Negative
	LD82	D25	MWD	Bouches-du-Rhône	10.2020	0.314	27.1	Negative	Not tested
	D417		MWD		02.2021	0.78	196.7	Positive	**Positive**
	D660	D26	PD		11.2020	0.049	14.5	Doubtful	**Positive**
	D661		PD		12.2020	0.105	38.3	Positive	**Positive**
Dogs collected three times, once before and twice during the pandemic (*N* = 11)	LD84	D28	MWD	Bouches-du-Rhône	06.2018	0.101	3.7	Negative	Not tested
	LD83		MWD		10.2020	1.503	157.6	Positive	**Positive**
	D408		MWD		02.2021	0.512	123.1	Positive	**Positive**
	LD86	D29	MWD		06.2018	0.111	4.8	Negative	Not tested
	LD85		MWD		10.2020	0.592	57.6	Doubtful	**Positive**
	D382		MWD		02.2021	0.549	133.2	Positive	**Positive**
Negative controls	D569	D30	MWD	Lot	02.2021			Negative	Negative
	D570	D31	MWD		02.2021			Negative	Negative
	D571	D32	MWD		02.2021			Negative	Negative

SD: shelter dog, MWD: military working dog, PD: pet dog, OD: optical density, AWB: automated western-blot.

**Table 3:** Comparison of seroprevalences (ELISA) of SARS-CoV-2 infection in dogs from the French departments of Bouches-du-Rhône (South-East) and Lot (South-West) in February 2021, and the correlation with the COVID-19 incidence in humans.

Location department	No. of dogs	No. of ELISAnegative dogs	No. of ELISA-positive dogs	Canine seroprevalence (%)	Incidence rate human COVID-19 per 100,000 inhabitants as of 02/16/2021	Seropositivity rate of human COVID-19 (%) as of 02/16/2021
Bouches-du-Rhône (South-East)	126	113	14	11.1	332	6.6
Lot (South-West)	95	92	3	3.1	100	2.9
Total	221	205	17	7.7		
Statistics				Khi2 = 4.7, p ≤ 0.05	Khi2 = 124, p ≤ 0.001	

### 3.2 Automated western blot results

**Fig. 1:**
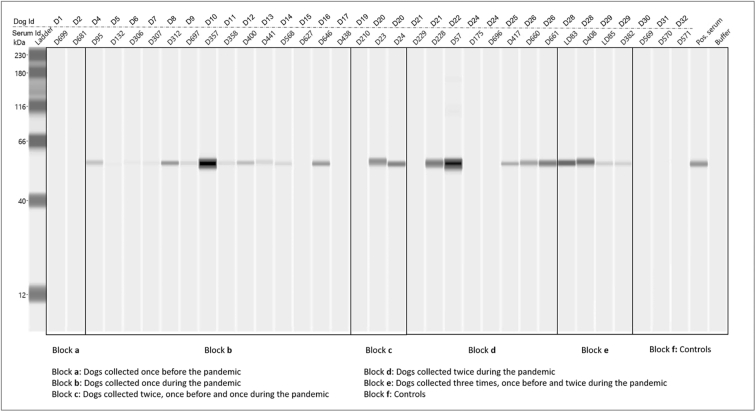
Results of the automated western blotting assay of SARS-CoV-2 infection in dogs from France, before and during the COVID-19 pandemic (*N* = 32).

Of the selected 44 serum samples listed in Table 2, 34 (consisting of 25 positive, 3 doubtful and 6 negative sera by ELISA assay). AWB detected 17 (68%) of the 25 ELISA-positive sera herein tested. In addition, all doubtful sera within ELISA test (*n* = 3) were scored positive by the AWB assay. Overall, all AWB-positive sera were sampled between the period ranging from January 2020 to February 2021. While no ELISA-positive sera collected before the pandemic or negative controls were detected by the AWB (Fig. 1). Which correspond to a significant difference between these two assays (Q = 8, *p* = 0.008). Finally, all AWB-positive sera yielded a prominent 56-kDa band interpreted as nucleocapsid proteins, while no bands were detected for the other major dominant proteins, such as the protein S (i.e. 170 kDa), S1 (i.e. 110 kDa) and S2 (i.e. 90 kDa) (Fig. 1).

## 4 Discussion

To date, studies investigating SARS-CoV-2 in dogs are scarce, probably due to the lower susceptibility of dogs to this infection and the focus of research on human disease. In France, only two serological studies have been carried out on dogs. One study based on the luciferase immuno-precipitation assay and involving 12 dogs from SARS-CoV-2-positive owners. None of these dogs was scored positive [18]. The second study was performed using microsphere immunoassay on 13 dogs from SARS-CoV-2-positive owners and 22 dogs from owners with an unknowing SARS-CoV-2 status. Only two dogs were reported positive by the authors from positive owners [19]. In Italy, the antibody neutralization assay was used to monitor SARS-CoV-2 infection in 451 dogs during the pandemic, and 15 (3.3%) dogs were found seropositive [20]. In Wuhan city (China), 16 (1.7%) positive dogs were detected among the 946 using a newly developed double-antigen sandwich ELISA assay [21]. In Croatia, an investigation revealed that 7.6% of dogs (13/172) were positive by ELISA assay [22]. In Texas, USA, 15.3% of 59 dogs were positive for SARS-CoV-2 by RT-PCR and genome sequencing or neutralizing antibodies, in homes where at least one human case of COVID-19 was diagnosed [23]. The overal prevalence of canine SARS-Cov-2 infection in Spain was 16.7% (10/60), particularly in dogs from COVID-19-positive households, indicating their susceptibility to SARS-CoV-2 infection [24]. These discrepancies in results between the different studies may be related to the sensitivity of the different assays. The results of this comprehensive study of SARS-CoV-2 infection in companion and military working dogs sampled before and during the pandemic in areas of active human viral transmission allowed the evaluation of the specificity of the ELISA and AWB assays. The same ELISA test used in our study detected anti-SARS-CoV-2 antibodies in the serum of PCR-positive cats, living in a household in Chile, where a human was infected [25].

In our study, the ELISA test we used detected 1.1% of 449 pre-pandemic sera. This highlights the possible cross reactivity with other canine coronaviruses, probably the Betacoronavirus of dogs [26]. On the other hand, the seroconversion of 8, as well as the significant increase in seroprevalence in dogs during the pandemic (i.e., 5.5% out of 453 dogs tested), particularly in the Bouches-du-Rhône region, a high endemic area for human SARS-CoV-2 infection (www.cascoronavirus.fr), could explain the occurrence of SARS-CoV-2 infection in dogs. On the other hand, the AWB assay yielded the detection of 66% ELISA-positive sera. However, all of them were sampled between the periods ranging from January 2020 to February 2021, which is in line with the outbreak of the pandemic in France. In addition, some inconsistencies were also observed between these two assays. For example, some dogs with high ELISA S/P ratio sampled before the pandemic (i.e. dog D1 and D2) or even during the pandemic (i.e. dog D6 and D7) gave a negative AWB result, whereas some ELISA-negative or doubtful sera with low ELISA S/P ratio (i.e. dog D14, D22, D26 and D29) were positive using AWB assay (Fig. 1). Though few canine sera were herein tested by the AWB, which may represent a limitation of the assay, all AWB-positive sera were sampled during the pandemic which suggests the specific detection of antibodies to SARS-CoV-2 in dogs. The discrepancy between these two assays could be explained by the type of antigens used for each assay. ELISA test was developed on the basis of a truncated N recombinant antigen from the viral nucleocapsid which probably provided the detection of conformational epitopes that could also be shared with the other coronaviruses. In contrast, the use of the integral SARS-CoV-2 nucleocapsid antigens in AWB assay may led the detection of linear epitopes only [17]. However, the clear-cut decision regarding the specificity of the AWB assay cannot be ruled out in the absence of a reliable gold standard, since the possible cross-reaction has already been described with other human Betacoronavirus within the AWB assay [17].

The AWB assay based on the purified virus antigens was first adapted for the diagnosis and the evaluation of the human immune-response against SARS-CoV-2 antigens. The assay proved to be effective principally in detecting antibodies to nucleocapsid proteins [17]. Our results showed that the AWB yielded only the detection of antibodies against the nucleocapsid proteins from all positive dogs. However, the lower sensitivity of AWB to spike proteins in dogs may due to the use of the integral, which may give rise hidden epitopes.

Despite the receptivity of dogs to SARS-CoV-2 infection under experimental conditions [27], they were unable to transmit the virus [7,9,10]. Our results indicated that, in spite of the presence of positive dogs in kennels, there were most probably few infected animals. Thereby, this suggests that dogs do not transmit the virus, which may be due to the poor viral replication in dogs [28]. On the other hand, previous studies have demonstrated the presence of a few differences between human and canine angiotensin-converting enzyme 2 (ACE2), the interactive receptor within the spike protein of the SARS-CoV-2 [9]. However, recent studies have demonstrated the continuous emergence of new SARS-CoV-2 with multiple spike protein mutations. It is not known whether dogs infected with these new variants could transmit the virus to other animals or to humans [[29], [30], [31]]. In March 2021, a study carried out on British dogs reported for the first time canine and feline infections with the SARS-CoV-2 B.1.1.7 variant in addition to some of these pets suffering from myocarditis [32].

## 5 Conclusion

The AWB assay, previously standardized as first or second line method to confirm the diagnosis of SARS-CoV-2 from human patients, could also be used as a second line assay to confirm negative, doubtful and discrepant ELISA results in dogs. These findings along with the results from the previous experimental models of SARS-CoV-2 in dogs confirm the receptivity of dogs to SARS-CoV-2 infection. They also suggest the absence of the virus transmission from infected to non-infected dogs as well as to humans. In the absence of genomic data on SARS-CoV-2 in dogs, the hypothesis that new SARS-CoV-2 variants with multiple mutations in the spike protein could induce adaptation of the virus to dogs cannot be ruled out.

## Ethics approval and consent to participate

All applicable international, national and military guidelines for the care and use of dogs were followed. The owners of the dogs have given their consent for the samples to be taken.

## Funding

This study was supported by the Health Service of the French Army and the Institut Hospitalo-Universitaire (IHU) Méditerranée Infection, the National Research Agency under the program “Investissements d'avenir”, reference ANR-10-IAHU-03, the Region Provence-Alpes-Côte d'Azur and European funding FEDER PRIMI.

## Author statement

In French and European legislation, our study does not enter into the regulation of laboratory animals and protocols submitted to an ethics committee.

The dogs studied are working and companion dogs. We (doctors in veterinary medicine) took serum (2 mL) from these dogs as part of disease screening and epidemiological surveillance. We have obtained the agreement of the owners of the dogs. Samples are taken in veterinary clinics (military, shelters and private).

## Declaration of Competing Interest

There are no competing interests. Two authors (Loïc Comtet and Philippe Pourquier) are currently employees of Innovative Diagnostics company, however there were no conflicting interests that may have biased the work reported in this paper.

## Appendix A Supplementary data

Validation report for ELISA test provided by the custumer. Image 1
