# Supplementary material for: SARS-CoV-2 antibodies seroprevalence in dogs from France using ELISA and an automated western blotting assay
Source: One Health. 2021 Jul 18;13:100293. doi: 10.1016/j.onehlt.2021.100293 (PMC8327341; doi:10.1016/j.onehlt.2021.100293)
Supplement: Supplementary file 1 — Validation report for ELISA test provided by the custumer. [file mmc1.pdf]

INTERNAL VALIDATION REPORT

ID SCREEN® SARS-COV-2 DOUBLE ANTIGEN MULTI SPECIES

Double antigen ELISA for the detection of antibodies directed against the nucleocapsid of SARS-CoV-2 in animal serum, plasma or whole blood

FOR VETERINARY USE ONLY

METHOD	Double antigen ELISA	
TARGET	Antibodies against the nucleocapsid of SARS-CoV-2	
SAMPLE TYPES	• Serum (preferred)<br>• Plasma (preferred)<br>• Total whole blood (alternative sample)	
VALIDATED SPECIES	Cats, dogs, horses, bovine, ovine, goat, ferrets, and any other susceptible species	
PRODUCT CODES AND FORMATS	**COVIDA-2P**<br>192 tests	**COVIDA-5P**<br>480 tests

Table of contents

INTRODUCTION 3
DESCRIPTION AND PRINCIPLE OF THE TEST 3
SPECIFICITY 4
SENSITIVITY 6
EXCLUSIVITY 7
REPEATABILITY 8
REPRODUCIBILITY 9
ROBUSTNESS 10
STABILITY 11
CONCLUSION 12
RELATED PRODUCTS 12
ACKNOWLEDGMENTS 12
HISTORY OF REVISIONS 12

INTRODUCTION

The COVID-19 pandemic in humans has led to increased interest in determining the prevalence of SARS-CoV-2 in animal species. The virus has already been detected in cats [3] and in mink farms [2]. The OIE made a review of the susceptibility of 10 animal species to SARS-CoV-2 and their possibility to transmit the virus [4].

Scientists are proceeding to epidemiological surveys in a variety of species and require an accurate and easy-to-use serological test.

To meet this demand, IDvet has launched its double antigen ELISA based on the nucleocapsid to detect anti-SARS-CoV-2 antibodies in multiple species.

This document outlines the validation data obtained for this test.

DESCRIPTION AND PRINCIPLE OF THE TEST

Wells are coated with purified N protein recombinant antigen.

Samples to be tested and controls are added to the microwells. Anti-SARS-CoV-2 antibodies, if present, form an antibody-antigen complex.

A purified N protein recombinant antigen horseradish peroxidase (HRP) conjugate is added to the microwells. It fixes to the free Fab of the bound serum anti-SARS-CoV-2 antibodies.

After washing to eliminate the excess conjugate, the Substrate Solution (TMB) is added.

The resulting coloration depends on the quantity of specific antibodies present in the specimen to be tested:

- In the presence of antibodies, a blue coloration appears which becomes yellow after addition of the Stop Solution.
- In the absence of antibodies, no coloration appears.

The microplate is read at 450 nm.

For each sample, calculate the S/P % is calculated:

$$S/P\,\% = \frac{OD_{Sample} - OD_{NC}}{OD_{PC} - OD_{NC}} \text{ x } 100$$

RESULT	STATUS
**S/P% ≤ 50%**	**Negative**
**50% < S/P% < 60%**	**Doubtful**
**S/P% ≥ 60%**	**Positive**

Note: Positive results may be confirmed by other serological techniques (virus neutralization, indirect immunofluorescence).

SPECIFICITY

The following sera sampled in the pre-epidemic period were tested (at IDvet and IHU Marseille, France) * using the ID Screen® SARS-CoV-2 Double Antigen Multi-species ELISA:

- 314 dog sera,
- 92 cat sera,
- 88 cattle sera,
- 86 horse sera,
- 83 goat sera,
- 86 sheep sera,
- 39 wild animals sera (bat (n=15), agouti (n=1), opossum (n=1), ocelot (n=1), jaguar (n=1), camel (n=12), howler monkey (n=8)).

Results are shown in Figure 1 and Table 1 below.

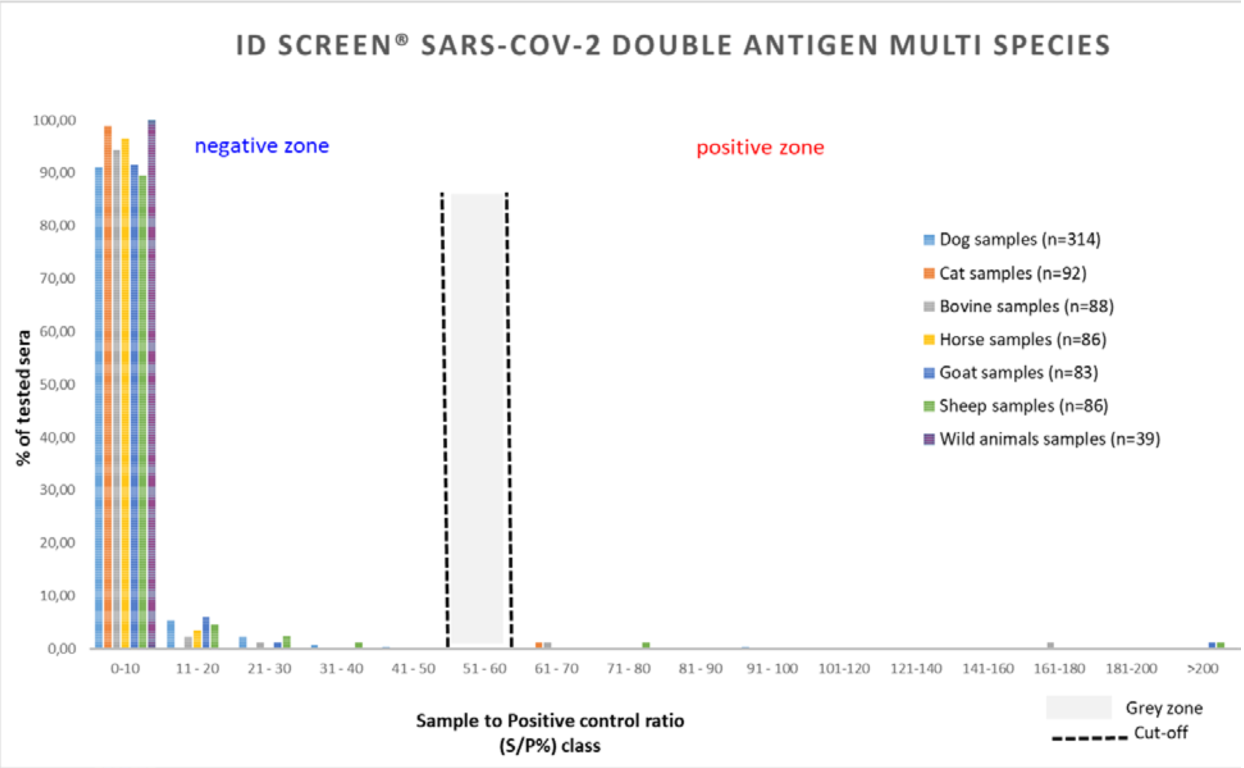

Figure 1: S/P% distribution for negative animals, n=788

SPECIES	SPECIFICITY (%) (number of samples tested)	$CI_{95\%}$
Horse	100 (n = 86)	95.7 - 100 %
Bovine	97.8 (n = 88)	92.1 - 99.4 %
Cat	98.9 (n = 92)	94.1 - 99.8 %
Dog	99.7 (n = 314)	98.2 - 99.9 %
Sheep	98.9 (n =86)	93.8 - 99.8 %
Goat	98.8 (n = 83)	93.5 - 99.8 %
Wild animals	100 (n = 39)	91.0 - 100 %
**TOTAL**	**99.1 (n = 788)**	**98.2 – 99.6 %**

Table 1: Measured specificity for negative animals, n=788

RESULTS (Figure 1 and Table 1) :

- Out of 788 sera, 781 sera were found negative.
- Measured specificity: 99.1 % ($CI_{95\%}$: 98.2 % - 99.6%), n = 788
- The ID Screen® ELISA shows a very good specificity.

SENSITIVITY

Sensitivity data is very limited since documented positive sera in animal species are not readily available.

▶ Please refer to the following publication [1], where a **cat sample** was found positive by ELISA and by RT-PCR:

Corinne Sailleau, Marine Dumarest, Jessica Vanhomwegen, et *al*. First detection and genome sequencing of SARS-CoV-2 in an infected cat in France. Authorea. May 19, 2020. DOI: 10.22541/au.158990358.89168563

For this study, the ID Screen® SARS-CoV-2-N IgG Indirect kit was used with a multi-species conjugate. Further studies, however, showed that this same cat serum was even more efficiently detected using the ID Screen® SARS-CoV-2 Double Antigen Multi-Species (COVIDA) ELISA test.

▶ **A sample from an experimentally infected ferret,** collected at 21 days post-infection, and found highly positive by both the indirect immunofluorescence antibody test (IFAT) and the Sero Neutralisation Test (SNT), was also tested on the ID Screen® SARS-CoV-2 Double Antigen Multi-species ELISA. This sample gave a high positive result with a S/P% of 95%, confirming susceptibility of ferrets to infection by SARS-CoV-2 [3].

▶ **8 minks with clinical cases,** from one infected farm, were sampled (whole blood and heparine plasma) and tested. The samples were negative with a SARS-CoV-2 qPCR but found positive for antibodies on an in-house ELISA (spike protein based).

All the samples tested were found positive, with a S/P value of 112 to 316% (median value: 195%).

Mink samples were also tested by serial dilutions demonstrating:

- a median titer of 1:8
- a lowest titer of 1:4
- a highest titer of 1:64

Results are shown in figure 2:

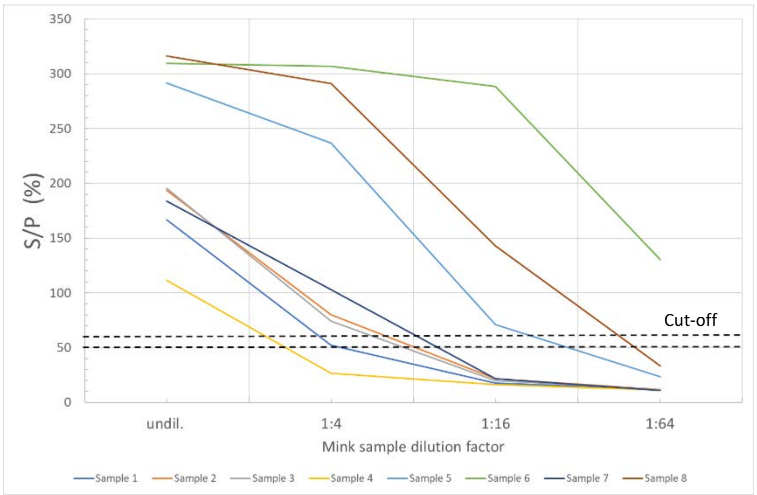

Figure 2: positive samples from mink in serial dilutions, n=8

RESULTS :

- All of the 10 infected animals were found positive with the ID Screen® ELISA.
- This test is able to detect different species such as cat, ferret and mink. For mink samples, plasma as well as whole blood were tested sucessfully.

EXCLUSIVITY

Cross-reactions with other coronavirus from animal species were measured on the ID Screen® ELISA using 38 samples collected in the pre-epidemic period. 30 sera from chickens infected with the Infectious Bronchitis Virus (IBV) and 8 sera from pigs infected with the Porcine Epidemic Diarrhea virus (PEDV) were tested.

Results are shown in Figure 3 below.

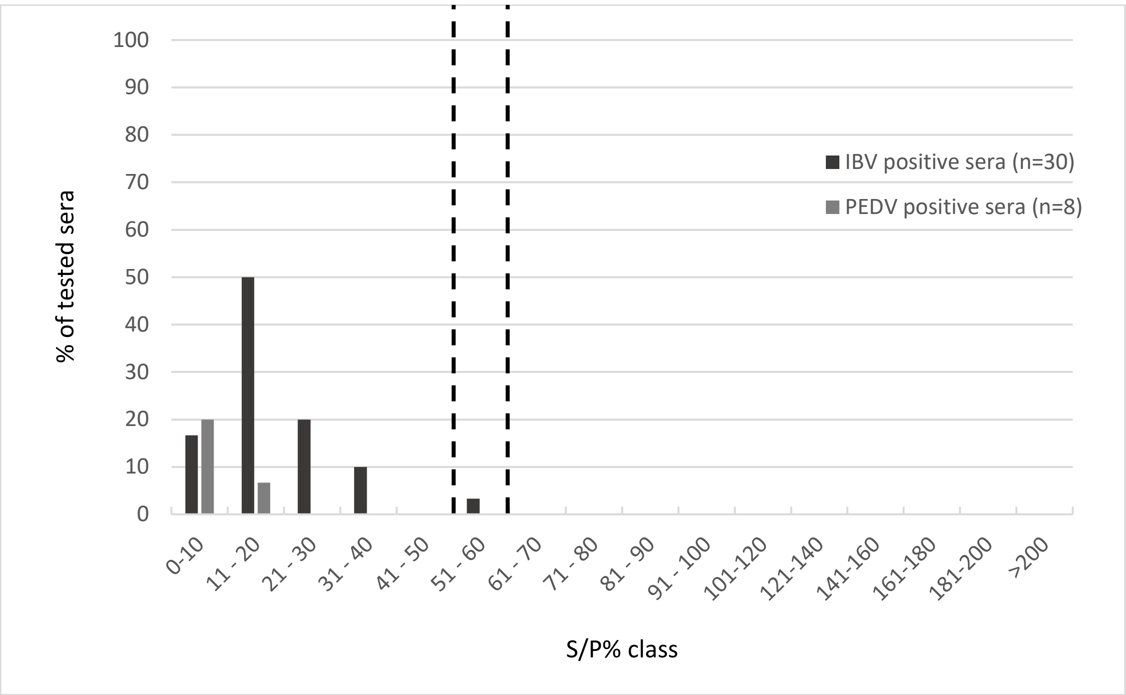

Figure 3: S/P% distribution for IBV infected chickens (n=30) and PEDV infected pigs (n=8).

RESULTS (Figure 3) :

- Out of 38 cross-reaction sera, 37 were found negative and only 1 was found doubtful with the ID Screen® ELISA.
- This test does not cross-react with other coronavirus such as IBV and PEDV, which means that the ID Screen® ELISA has a **very good exclusivity.**

REPEATABILITY

Intra-plate repeatability was evaluated by measuring the coefficient of variation (CV%) for 36 repetitions of a strong positive sample, and 60 repetitions of a weak positive sample.

Results are considered conform if the CV% is less than 10%. OD results are shown Table 2 below.

OD AT 450NM											
0.721	0.704	0.703	0.671	0.708	1.282	1.216	0.653	0.699	0.719	0.667	0.674
0.704	0.689	0.684	0.685	0.674	1.187	1.179	0.648	0.656	0.649	0.689	0.664
0.684	0.657	0.661	0.643	0.661	1.256	1.172	0.611	0.653	0.635	0.651	0.664
1.201	1.119	1.086	1.111	1.067	1.035	1.024	1.018	1.052	1.045	1.113	1.056
1.214	1.137	1.077	1.104	1.127	1.109	1.085	1.099	1.063	1.106	1.072	1.212
0.677	0.678	0.655	0.628	0.663	1.224	1.163	0.628	0.623	0.639	0.651	0.658
0.707	0.694	0.700	0.662	0.680	1.232	1.236	0.671	0.643	0.655	0.598	0.667
0.685	0.685	0.680	0.689	0.723	1.229	1.248	0.628	0.622	0.639	0.647	0.638

	AVERAGE OD	STANDARD DEVIATION	MINIMUM	MAXIMUM	CV%
Weak positive sample	0.667	0.028	0.598	0.723	4
Strong positive sample	1.138	0.075	1.018	1.282	7

Table 2: Repeatability study for the ID Screen® ELISA (results expressed as OD values).

RESULTS (Table 2) :

- The CV% obtained were 4% for the weak positive sample and 7% for the strong positive sample, demonstrating **excellent test repeatability**.

REPRODUCIBILITY

A positive serum was diluted in a pool of negative serum in order to generate a threshold sample.

This threshold dilution was tested in 25 independent runs by different operators and on different days.

Results are shown in Figure 4.

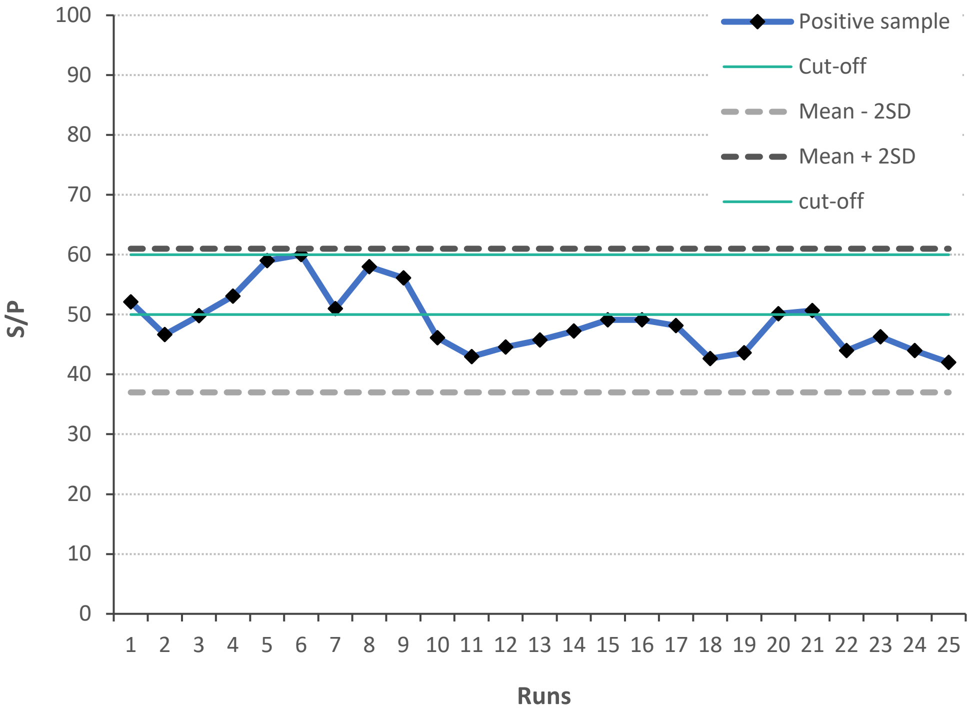

Figure 4: S/P values for a threshold dilution of a positive serum sample tested in 25 independent runs.

RESULTS (Figure 4) :

- **All values are within a range of 2 standard deviations around the mean, with a CV% of 11%.**
- These results illustrate the **high reproducibility of the ID Screen® ELISA test.**

ROBUSTNESS

- **Test robustness was evaluated by 3 operators in 3 independent runs.**

RESULTS:

For each run:

- **The validation criteria** described in the insert for both **positive and negative controls were obtained.**
- **S/P values for negative control, positive control and threshold samples were equivalent, regardless of the test conditions.**

- **Robustness was evaluated by testing the maximum and minimum conditions of time and temperature of incubation as defined in the instructions for use:**
  - Samples incubation: 45 minutes ± 5 minutes at 37°C (± 2°C);
  - Conjugate incubation: 30 minutes ± 3 minutes at 21°C (± 5°C);
  - Substrate Solution incubation: 20 minutes ± 2 minutes at 21°C (± 5°C).

For each condition. the test is validated if:

- The mean value of the Positive Control OD (OD_{PC}) is greater than 0.350 ($OD_{PC} > 0.350$).
- The ratio of the mean values of the Positive and Negative Controls (OD_{PC} and OD_{NC}) is greater than 3 ($OD_{PC}/OD_{NC} > 3$)

Optical densities at 450nm obtained in each condition for both negative and positive controls and the S/P% values obtained for 3 dilutions of a positive sample and 2 negative samples are detailed in Table 3 below.

SAMPLES/CONJUGATE/SUBSTRATE INCUBATION TIME	45 MIN / 30 MIN / 20 MIN			40 MIN / 27 MIN / 18 MIN	50 MIN / 33 MIN / 22 MIN	
TEMPERATURE OF INCUBATION OF SAMPLES	35°C	37°C	39°C	35°C	39°C	
TEMPERATURE OF INCUBATION OF CONJUGATE	16°C	21°C	26°C	16°C	26°C	
Negative control	0.059	0.060	0.061	0.052	0.069	OD 450 NM
	0.054	0.065	0.058	0.056	0.081	
Positive control	0.938	0.979	1.410	0.819	1.450	
	0.982	1.009	1.339	0.808	1.382	
**$OD_{NC} \leq 0.150$**	√	√	√	√	√	
**$OD_{PC} - OD_{NC} \geq 0.150$**	√	√	√	√	√	
Positive sera pure	180	162	164	182	166	S/P%
Positive sera diluted 1:2	95	92	100	87	104	
Positive sera diluted 1:4	49	51	59	44	59	
Negative sample 1	7	8	7	5	8	
Negative sample 2	8	9	10	6	9	

Table 3: Robustness study for the ID Screen® ELISA

RESULTS (Table 3):

- For each time and temperature condition, the test validation criteria for both positive and negative controls were obtained.
- For each time and temperature condition, the S/P values obtained were similar, and analytical sensitivity was constant, thereby demonstrating the **excellent robustness** of the ID Screen® ELISA.

STABILITY

The shelf-life of the products is evaluated by the technique of accelerated ageing.

The stability of the plates, the positive control and the conjugate was tested by evaluating the residual activity of individual components after storage at 37°C ± 2°C, with respect to storage at 5°C ± 3°C. The measured residual activity at 37°C ± 2°C should be greater than 75% after two months.

Results are shown in Figure 5 below.

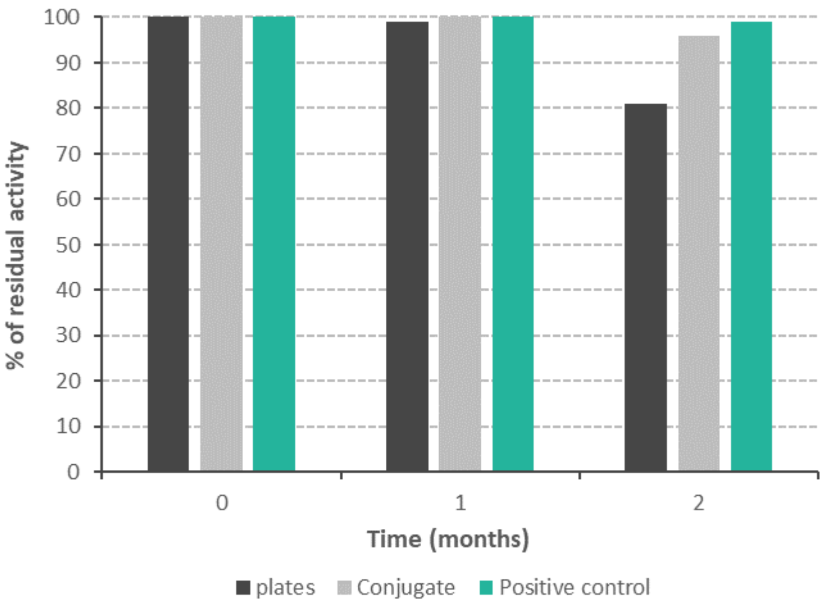

Figure 5: Percentage of residual activity of the plates. positive control and conjugate after stability testing at 37°C.

RESULTS (Figure 5):

- After 2 months at 37°C, the plates, the conjugate and the positive control showed residual activity of 81%, 96% and 99% respectively, thus indicating **high component stability.**

CONCLUSION

The **ID Screen® SARS-CoV-2 Double Antigen Multi-species ELISA** shows high specificity in a number of animal species, and successfully detected a RT-PCR positive cat serum [1], a IFAT/SNT positive ferret serum and in house ELISA positive minks whole blood and plasma samples.
The **ID Screen® SARS-CoV-2 Double Antigen Multi-species ELISA** demonstrates high exclusivity against avian and porcine coronaviruses. No cross-reactivity with other coronaviruses (such as feline or canine coronaviruses) haven't been tested yet, due to the absence of characterized samples. However, due to the protein N sequence, no or very limited cross-reaction should be expected in other animal coronaviruses.

References

(1) Corinne Sailleau, Marine Dumarest, Jessica Vanhomwegen, et al. **First detection and genome sequencing of SARS-CoV-2 in an infected cat in France**. Authorea. May 19, 2020.
DOI: 10.22541/au.158990358.89168563

(2) **CORONAVIRUS DISEASE 2019 UPDATE (174): Netherlands (North Brabant) Animal, Farmed Mink, Comment**. PROMED post May 11th, 2020.

(3) J. Shi et al., **Susceptibility of ferrets, cats, dogs, and other domesticated animals to SARS–coronavirus 2**, Science 10.1126/science.abb7015 (2020).

(4) **Infection with sars-cov-2 in animals**, OIE TECHNICAL FACTSHEET, version updated July 2020.

Related products

For associated products, please consult the IDvet website: www.id-vet.com

Acknowledgments

* IDvet would like to thank for their contribution to the specificity evaluation:

- B. Davoust et Y. Laidoudi (IHU Méditerranée Infection, Marseille, France), personnal communication.

History of revisions

VERSION	EDIT DATE	REFERENCE	TYPE OF REVISION	REVISION MADE
0520	01/2021	DOC981	Update : Addition/Edition of validation data	Addition of specificity data: dog, cat and widl animal samples. Addition of acknowledgments section.
	11/2020	DOC965	Correction of anomalies in the document	Correction of references numbers in introduction and conclusion
	10/2020	DOC943	Update : Addition/Edition of validation data	Addition of a new sample type : total blood<br>Addition of repeatability, robustness, stability, exclusivity.<br>Modification of specificty and sensitivity : data obtained on batch PT052
	07/2020	DOC892	Update : Addition/Edition of validation data	Additional information on the ferret sample tested.
	06/2020	DOC881	Not applicable (first version)	N/A
